# Monolithic integration of visible GaAs and near-infrared InGaAs for multicolor photodetectors by using high-throughput epitaxial lift-off toward high-resolution imaging systems

**DOI:** 10.1038/s41598-019-55159-x

**Published:** 2019-12-09

**Authors:** Dae-Myeong Geum, SangHyeon Kim, Seong Kwang Kim, SooSeok Kang, JiHoon Kyhm, Jindong Song, Won Jun Choi, Euijoon Yoon

**Affiliations:** 1 Department of Materials Science and Engineering, Seoul National University, Gwanak-ro 1, Gwanak-gu, Seoul 08826 Republic of Korea; 2 Center for Opto-Electronics Materials and Devices, Korea Institute of Science and Technology (KIST), Hwarangno 14-gil 5, Seongbuk-gu, Seoul 02792 Republic of Korea; 3 School of Electrical Engineering, Korea Advanced Institute of Science and Technology (KAIST), Daehak-ro 291, Yuseong-gu, Daejeon 34141 Republic of Korea; 4 Research Institute of Advanced Materials & Inter-university Semiconductor Research Center, Seoul National University, Gwanak-ro 1, Gwanak-gu, Seoul 08826 Republic of Korea; 5 Quantum Functional Semiconductor Research Center, Dongguk University, Joong-gu, Seoul 04620 Republic of Korea

**Keywords:** Electrical and electronic engineering, Optoelectronic devices and components

## Abstract

In this study, multicolor photodetectors (PDs) fabricated by using bulk p-i-n-based visible GaAs and near-infrared InGaAs structures were monolithically integrated through a high-throughput epitaxial lift-off (ELO) process. To perform multicolor detection in integrated structures, GaAs PDs were transferred onto InGaAs PDs by using a Y_2_O_3_ bonding layer to simultaneously detect visible and near-infrared photons and minimize the optical loss. As a result, it was found that the GaAs top PD and InGaAs bottom PD were vertically aligned without tilting in x-ray diffraction (XRD) measurement. A negligible change in the dark currents for each PD was observed in comparison with reference PDs through electrical characterization. Furthermore, through optical measurements and simulation, photoresponses were clearly revealed in the visible and near-infrared band for the material’s absorption region, respectively. Finally, we demonstrated the simultaneous multicolor detection of the visible and near-infrared region,which implies individual access to each PD without mutual interference. These results are a significant improvement for the fabrication of multicolor PDs that enables the formation of bulk-based multicolor PDs on a single substrate with a high pixel density and nearly perfect vertical alignment for high-resolution multicolor imaging.

## Introduction

Multicolor detection (or multispectral detection) of visible and infrared (IR) wavelengths has been widely employed for sensing and imaging applications, such as gas detection, medical diagnostics, wavelength-division multiplexing (WDM) and industrial surveillance systems^1,2^. In recent years, the emergence of autonomous cars in the internet-of-things (IoT) era has increased the demand for various sensors that can visualize objects with high accuracy in the light and night vision^3^. Additionally, visible/near-IR wavelength detection provides many new applications, such as the normalized vegetation differential index (NVDI) and time-of-flight (TOF) sensors for three-dimensional imaging^4,5^. Thus, regarding the recent development trend of integrating multi-functionality into a single chip, compact and low-power-consuming multicolor photodetectors (PDs) are urgently required^6^.

Among the several candidate materials to facilitate multicolor detection, groups IV, III-V semiconductors have been representative. While group IV materials, such as silicon (Si) and germanium (Ge), are suitable for cost-effective and large-area production, they can only tailor the wavelength to the near-IR region due to their inherent band gaps. On the other hand, III-V compound semiconductors are more promising materials because the detection wavelength can be extended from ultraviolet (UV) to the IR region with an appropriate material composition. Furthermore, the high mobility and direct bandgap of III-V materials are very suitable for the fabrication of PDs, which have high speed and high quantum efficiency.

Thus, to realize III-V-based multicolor PDs, many research groups have sought to epitaxially grow type-II superlattices and GaSb/InAsSb nBnBn bulk detectors with multiple sensing regions^7,8^. These type-II superlattices consist of multiple absorption regions that can be tuned by applying voltage. However, voltage-controlled behavior is difficult for simultaneous sensing regarding different wavelengths. Moreover, this can inevitably lead to high-power consumption. While a GaSb/InAsSb nBnBn bulk structure was formed on a GaAs substrate by using the interfacial misfit array (IMF) technique, a high dark-current density still exists due to defects and dislocations despite the insertion of a barrier layer. Furthermore, in both cases a discrepancy of lattices between the substrate and epitaxial layers defined as the critical thickness is inevitable.

Recently, to circumvent these epitaxy issues, heterogeneous integration by three-dimensional stacking has been extensively investigated as an approach to implement multicolor PDs as well as light-emitting diodes (LEDs) and metal-oxide semiconductor field-effect transistors (MOSFETs)^8–11^. The monolithic integration of various PDs has an important advantage for future multi-functional PDs. First, it could alleviate the system volume for multicolor detection, which results in compact and low-power-consuming detection systems^12^. Secondly, thanks to freedom of choice about stacking materials, heterogeneously integrated multicolor PDs enable the detection of various wavelengths with a variety of materials having UV, visible, and IR regarding target applications with high performance.

Although there are many advantages, few results of monolithic integration have been reported even in a combination of visible and near-IR wavelength^11,13^. Seo *et al*. demonstrated that the heterogeneous integration of a GaAs metal-semiconductor-metal (MSM) PD and an InGaAs MSM PD on a Si substrate by using a BCB adhesive bonding layer^12^. Each PD was vertically integrated and operated in its absorption regions. However, this method easily induced vertical and horizontal misalignments and required delicate post-processing conditions for adhesive bonding when they were combined^14^. Menon *et al*. presented InGaAs and Si membrane four-color PD arrays fabricated by a transfer printing method^13^. Thin-film Si PDs were transferred onto the plane of a top InGaAs PD grown on an InP substrate. Its structure consisted of an InGaAs PD next to Si PDs on the same plane. Although this planar integrated strcture could detect different wavelengths by using the Si and InGaAs PDs, the resolution and misalignment in the large-area process need to be improved. Therefore, top-down fabrication processes after monolithic integration are necessary to obtain high resolution and multi-functionalities.

Ultimately, to form multicolor PDs, the key technology is how to vertically incorporate the PDs without misalignment and performance degradations between top PDs and bottom PDs because it is very important that the single-pixel area is reduced compared with planar integration by 1/n, where n means the number of desired materials. Actually, according to the development trend of photodetectors even in light-emitting diodes (LEDs), many researchers have extensively investigated ways of making vertically stacked devices to obtain ultra-high-resolution imaging devices^15^. From this point of view, high-throughput epitaxial lift-off (ELO) was suggested as a feasible option in our previous article^16^. In comparison with hydrogen splitting called ‘smart cut’ and transfer printing, the high-throughput ELO approach has several advantages, such as reliability, throughput, and good epitaxial quality. In comparison to growth-based integration, which limits the material applicability due to lattice-mismatch, the wafer-bonding-based technique can be extended to any semiconductor materials, such as GaN, GaAs, InGaAs, InAs, InSb, and even group IV materials, without concerning lattice mismatch. Another important issue is to select the structure of the PDs itself. Among many PD structures, such as MSM and avalanche photodiodes (APDs), a simple p-i-n structure is promising because the photovoltaic mode contributes to high speed and low power consumption^17^.

**Figure 1:**
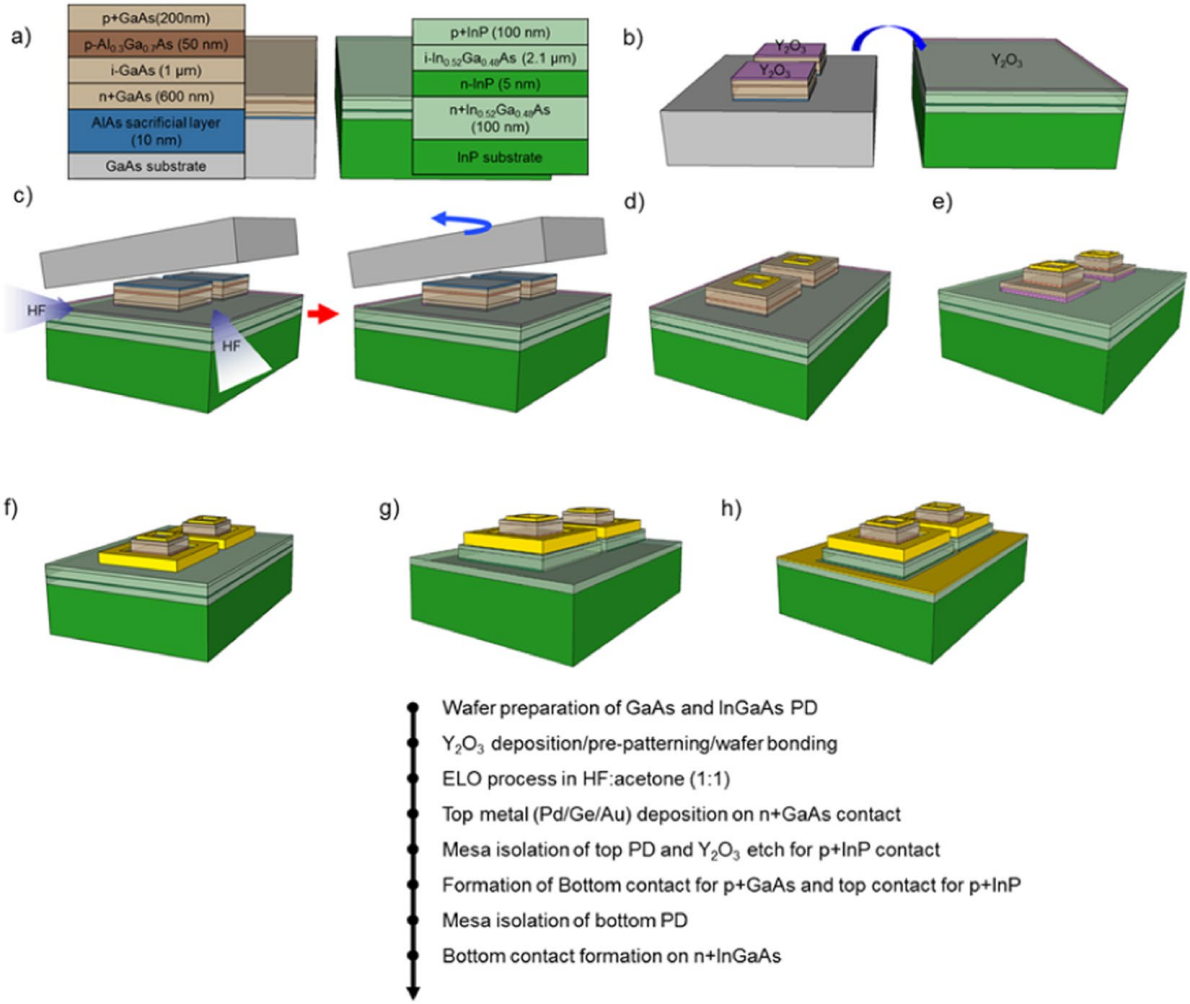
Fabrication process of multicolor PDs with Y_2_O_3_ bonding layer. Schematic of a process flow for GaAs//InGaAs p-i-n based multicolor PDs.

Therefore, in this study, we demonstrated the monolithic integration of p-i-n GaAs PDs to InGaAs PDs on a single substrate by using the high-throughput ELO method. After a GaAs-based p-i-n and InGaAs p-i-n bulk structure was grown on each substrate, multicolor PDs with three terminals (top/middle common/bottom contact) were formed on the InP substrate as shown in Fig. 1. A three-dimensional stacked PD makes it possible to simultaneously detect visible and near-IR wavelengths. Also, each pixel is vertically aligned from the top PD to the bottom PD, which enables the top-down fabrication process. The material quality of the fabricated multicolor PD was characterized by X-ray diffraction (XRD) measurement and transmission electron microscopy (TEM). The current-voltage (I-V) measurements clearly showed the diode characteristics of GaAs and InGaAs depending on the bias. Also, a clear photoresponse and responsivity was observed in terms of incident wavelengths in external quantum efficiency (EQE) and optical measurements. These optical properties of multicolor PD were consistent with the simulated optical intensity distribution. These results confirmed the feasibility of multicolor PD fabrication using simple bulk structures.

## Method

### Fabrication of multicolor PDs

Figure 1(a) shows the epitaxial structures of multicolor PDs consisting of the GaAs p-i-n structure and In_0.52_Ga_0.48_As p-i-n structure. The GaAs p-i-n structure was grown on a semi-insulating (S.I) GaAs substrate by using molecular beam epitaxy (MBE) forming an AlAs sacrificial layer (10 nm)/n + GaAs layer (600 nm)/intrinsic GaAs absorption layer (1 μm)/p + Al_0.3_Ga_0.7_As layer (50 nm)/p + GaAs layer (200 nm), from the bottom to top. The In_0.52_Ga_0.48_As structure grown on an S.I InP substrate consisted of an n + In_0.52_Ga_0.48_As bottom layer (200 nm)/n-InP etch stop layer (5 nm)/intrinsic In_0.52_Ga_0.48_As absorption layer (2.2 μm)/p-InP top layer (100 nm), which was formed by metal-organic chemical vapor deposition (MOCVD). The fabrication process started with a standard wafer cleaning process with acetone, methanol, and isopropyl alcohol (IPA). After the cleaning process, a 20-nm-thick Y_2_O_3_ layer was deposited on the top surfaces of both GaAs and In_0.52_Ga_0.48_As as a bonding material by electron beam (E-beam) evaporation. After deposition of the Y_2_O_3_ layer, as shown in Fig. 1(b), the shape of the top GaAs PDs was defined by photolithography and etched to the GaAs substrate with the exposed sacrificial layer by H_3_PO_4_-based solutions for the high-throughput ELO process. Then both Y_2_O_3_ surfaces were irradiated by oxygen plasma for surface activation, followed by wafer bonding at room temperature with a pressure of 3.4 MPa in a wafer bonder. The sample was put in hydrofluoric acid (HF): acetone (1:1) solutions to separate the GaAs substrate as shown in Fig. 1(c). When the GaAs substrate was separated, we obtained the vertically integrated GaAs n-i-p PD on InGaAs p-i-n PD structure. After formation of the integrated structure, typical PD fabrication processes were adopted for both PDs. The top electrode of Pd/Ge/Au (20/40/200 nm) was deposited on the n + GaAs layer by E-beam evaporation and a subsequent lift-off process as shown in Fig. 1(d). Mesa isolation of the top PD to the depth of the p + GaAs layer was carried out by using an H_3_PO_4_-based solution. Simultaneously, for the metallization of the common p-contact (middle contact), the Y_2_O_3_ interlayer was removed to expose the p-InP contact layer as seen in Fig. 1(e). Figure 1(f) shows the formation of the bottom contact of the top PD and the top contact of the bottom PD with Pt/Ti/Pt/Au (20/30/20/200 nm) metallization and lift-off. Then, mesa etching to the depth of the n + InGaAs layer for bottom PD isolation was conducted by using an H_3_PO_4_-based solution. Finally, as shown in Fig. 1(h), the bottom contact of Pd/Ge/Au (20/40/200 nm) for the n + InGaAs layer was formed, followed by rapid thermal annealing (RTA) at 350 °C for 1 min in N_2_ ambient.

## Results and Discussion

**Figure 2:**
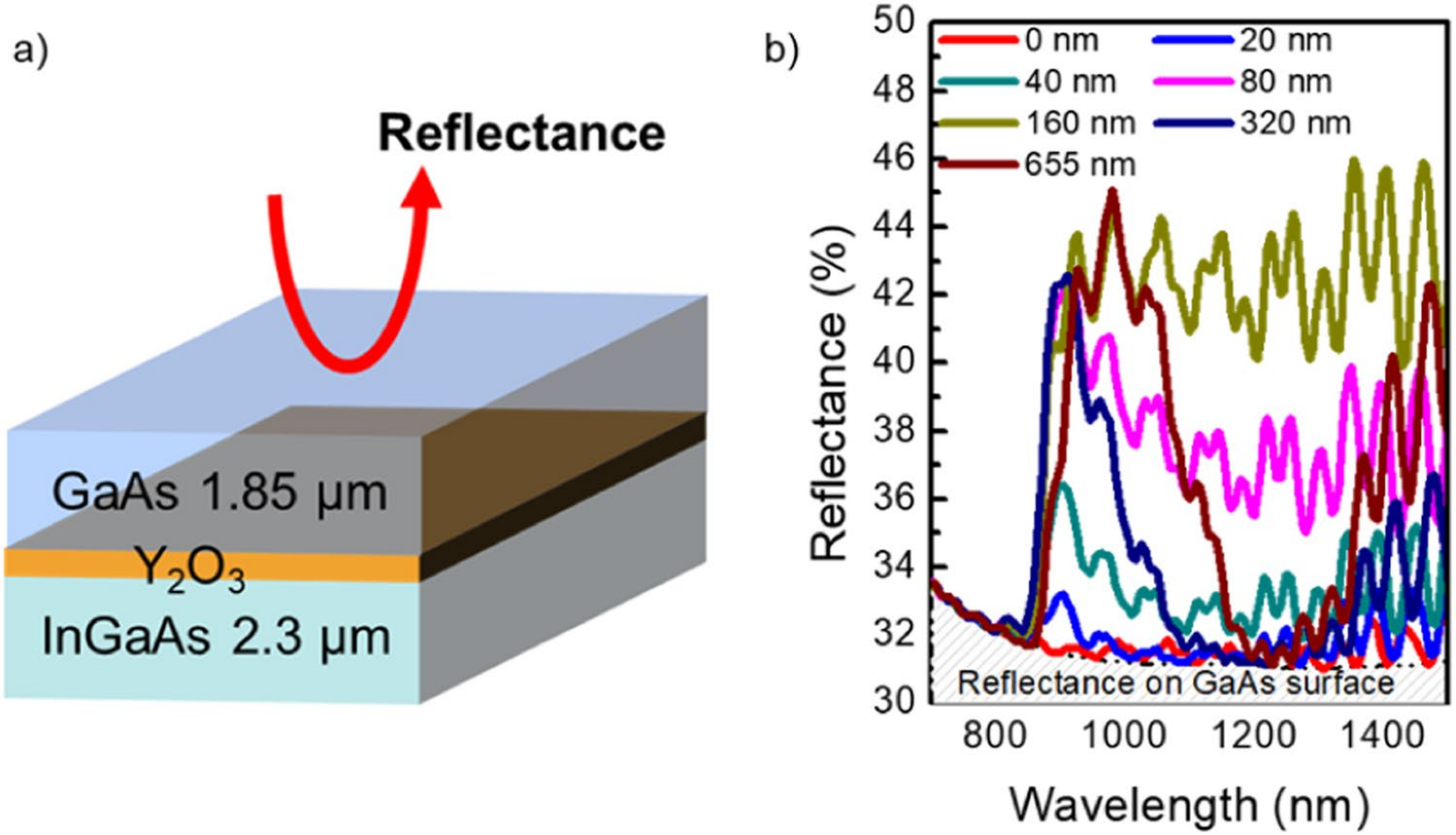
Investigation for effects of Y_2_O_3_ thickness variation on reflectance. (**a**) Schematic of the brief structure for the simulation (**b**) simulation results with different Y_2_O_3_ bonding layer thicknesses.

For the fabrication of multicolor PDs, as depicted in Fig. 1, the important reason for the use of Y_2_O_3_ as a bonding material is its high transmittance over a wide wavelength range from 400 nm to 13 μm, which is the best choice to alleviate the optical loss of multicolor PDs^18,19^. In addition, we investigated the effect of Y_2_O_3_ thickness on optical loss for this device scheme because the difference of refractive index between materials can induce internal reflection at interfaces. To assess the effects of Y_2_O_3_ thickness, a simple structure consisting of GaAs, Y_2_O_3_, and InGaAs with the same thickness as that of actual devices was used for a simulation as shown in Fig. 2(a). Figure 2(b) shows the resulting reflectance as a function of wavelength from 700 nm to 1500 nm. Fundamentally, the amount of reflected light was found to be 30% at the GaAs top surface, which is quite reasonable considering its refractive index of approximately 3.3. In the overall wavelength range, a high reflectance was observed with increasing Y_2_O_3_ thickness from 0 nm to 655 nm. Additionally, a thick Y_2_O_3_ layer could induce optical interference in the long-wavelength region due to the formation of a cavity structure. Due to this property, reflectance could be minimized at a specific wavelength near 1300 nm, when the Y_2_O_3_ bonding layer was 320 nm and 655 nm thick. Nevertheless, it should be ultimately decreased to diminish the optical loss at the material’s interface. Thus, considering both bonding conditions as well as optical loss, we chose the 40-nm-thick Y_2_O_3_ layer, which can be further reduced with the optimized bonding process.

**Figure 3:**
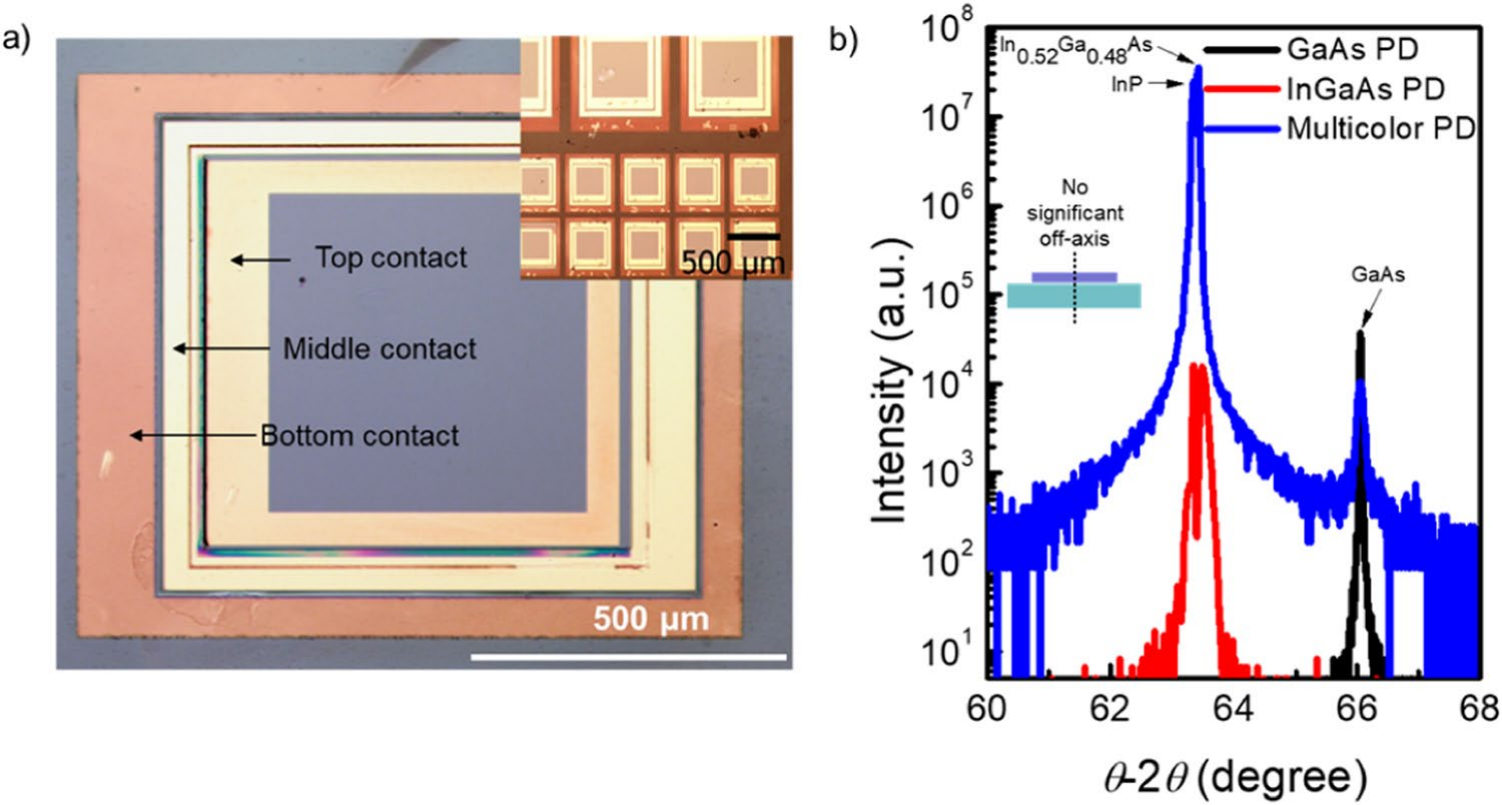
Characterization of fabricated multicolor PDs. (**a**) Optical image of multicolor PD with three terminals (top view), optical image for highly aligned 2 × 5 array (**b**) *θ-*2*θ* measurements for reference GaAs, InGaAs PD, and multicolor PD.

Figure 3(a) shows an optical microscope image of the top view of the fabricated multicolor PD with three terminals. Each electrode is clearly separated along the mesa edge without damaged regions. As shown in the inset figure of the optical image, a 2 × 5 multicolor PD array was successfully formed of top PDs and bottom PDs in the single-pixel region. Moreover, to accurately evaluate the crystalline qualities, high-resolution XRD (HRXRD) measurement was performed using an ATX-G equipped with Cu K_α1_ radiation and double crystals. Figure 3(b) shows the *θ*−2*θ* scan results for the InGaAs PD and GaAs PD as a reference and multicolor PD, respectively. Only one peak was observed for the GaAs PD at 66.05°, which corresponds to the GaAs (004) peak, while two peaks were observed for the InGaAs PD at 63.34° and 63.45° which correspond to the InP (004) InGaAs (004) peaks, respectively. Then, the XRD result of the multicolor PD showed a combination of GaAs PD and InGaAs PD peaks at 63.34°, 63.45°, and 66.05° which is responsible for substrate and contact layer of InP (004), InGaAs (004), and the thin film of GaAs (004), respectively. These same peak positions compared with the reference PD indicate that the GaAs PD was successfully integrated to the InGaAs PD without strain and severe offset of the vertical axis. Furthermore, the full-width at half-maximum (FWHM) values of the InGaAs and GaAs peaks were 167 arcsec and 373 arcsec, respectively. This suggests that the qualities of GaAs and InGaAs PDs would be maintained during the high-throughput ELO process and fabrication process.

**Figure 4:**
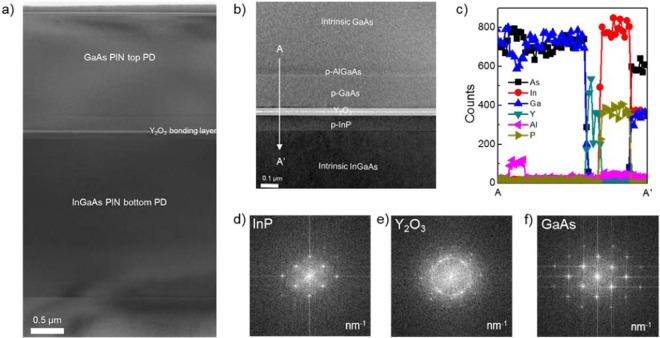
Investigation on bonding quality and material qualities for formed multicolor PDs structure. (**a**) cross-sectional TEM image with low magnitude for multicolor photodetectors, (**b**) expanded TEM image at the Y_2_O_3_ bonding interface, (**c**) EDAX profile in terms of each atom from A to A’, (**d**–**f**) fast Fourier transform (FFT) patterns of InP, Y_2_O_3_ and GaAs, respectively.

Figure 4(a) shows a cross-sectional transmission electron microscopy (TEM) image. It was noted that the GaAs PD was bonded onto the InGaAs PD with good uniformity and without any voids at the bonding interface. Also, there were no visible defects or dislocations in the active layer and surfaces of either PD. Figure 4(b) shows a high-resolution image obtained near the bonding interface. The abrupt interfaces of GaAs, AlGaAs, Y_2_O_3_, InP, and InGaAs were confirmed despite the thermal annealing process. The abrupt interfaces were also confirmed by energy dispersive x-ray (EDX) spectroscopy. Figure 4(c) depicts the measured energy dispersive spectroscopy (EDX) line profile from A to A’, which definitely shows boundaries of the Y_2_O_3_ bonding layer and epitaxial layers without inter-diffusion. While there is a concern regarding inter-diffusion near different materials for growth-based methods, bonding-based integration is free from diffusion issues because the Y_2_O_3_ layer plays an important role as the diffusion barrier as well as a bonding material. Figure 4(d–e) show the fast Fourier transform (FFT) patterns for InP, Y_2_O_3_ and GaAs, respectively. The patterns of InP and GaAs strongly suggest that good single-crystalline quality was sustained through wafer bonding, ELO, and even rapid thermal annealing (RTA). For the Y_2_O_3_ layer, its pattern indicates polycrystalline characteristics caused by partial crystallization of oxides in the RTA process, as seen in our previous works^20^. These results indicate that we successfully fabricated the multicolor PD in a single-pixel area without any material degradation. Therefore, this approach to monolithically integrate each PD in single pixels completely permits multicolor PDs to have high material qualities, process reliability, and no off-axis tilt along the out-of-plane direction.

**Figure 5:**
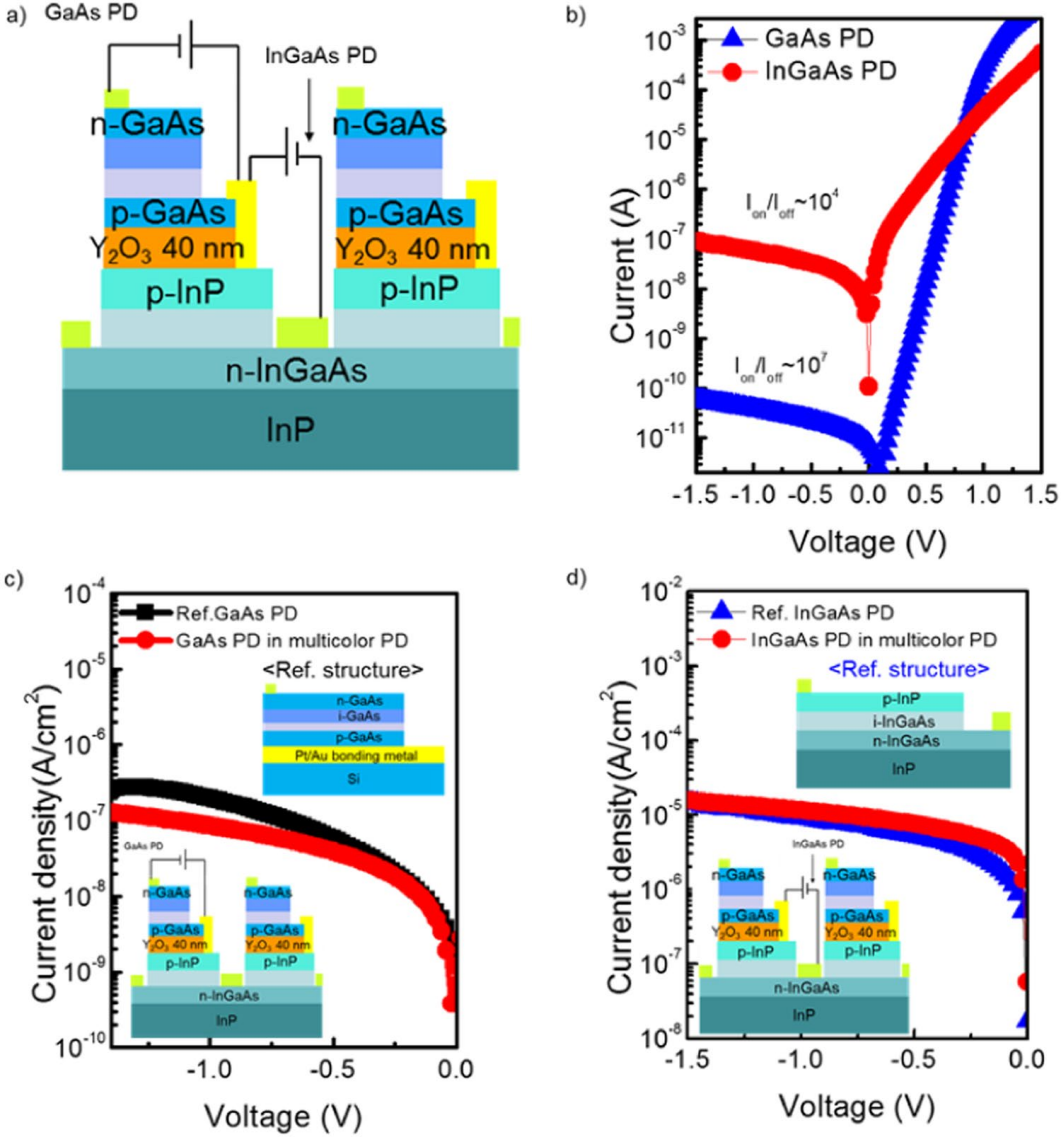
Electrical measurements for fabricated PDs for investigating the independent operation and process reliability. (**a**) schematic of final structure for electrical measurements of multicolor PDs, (**b**) GaAs and InGaAs PDs current-voltage in multicolor PD, (**c)** and **(d**) comparison of dark current between GaAs and InGaAs PD of reference structure and multicolor structure.

To investigate the electrical characteristics of multicolor PDs, the current (*I*) - voltage (*V*) characteristics of the fabricated devices were measured at room temperature as shown in the schematic of Fig. 5(a). Measurements were conducted by a Keithley 4200 in a probe station. The top GaAs PD had the dimensions of 335 × 315 μm^2^ and the bottom InGaAs PD had the dimensions of 425 × 375 μm^2^. Clear rectifying characteristics of each PD were found when bias from −1.5 V to 1.5 V was applied as seen in Fig. 5(b). While the GaAs PD showed an I_±1.5V_ ratio of approximately 10^7^ for a ± 1.5 V bias, that of the InGaAs PD showed approximately 10^4^ orders of magnitude. We measured 12 identical devices, which showed similar dark current levels and I_±1.5V_ ratios for the same bias range.

To compare the performance of the reference and multicolor PDs, we measured the dark current properties as shown in Fig. 5(c,d). Reference PD structures are shown in the figure. To equivalently compare performance in the dark current region of the GaAs top PD, the same transferred structure using GaAs PD on a Si substrate was plotted. When we assessed the leakage current between the GaAs top PD in the multicolor PD and the GaAs reference PD, the current density showed a similar level of 10^−7^ A/cm^2^ for both PDs for the whole bias range. No noticeable difference between the two PDs could be observed considering a slight device-to-device variation. The same comparison was performed for the reference InGaAs PD and the bottom InGaAs PD. The reference InGaAs PD was fabricated by using a typical PD process with the same metallization schemes and RTA process as that used to fabricate the multicolor PD. Although the multicolor PD went through the ELO process in an HF-based solution, no excess dark current was exhibited in comparison with the reference InGaAs PD. Verification of the I-V behaviors of the multicolor PD demonstrates that our fabrication process is viable to make high-performance devices.

**Figure 6:**
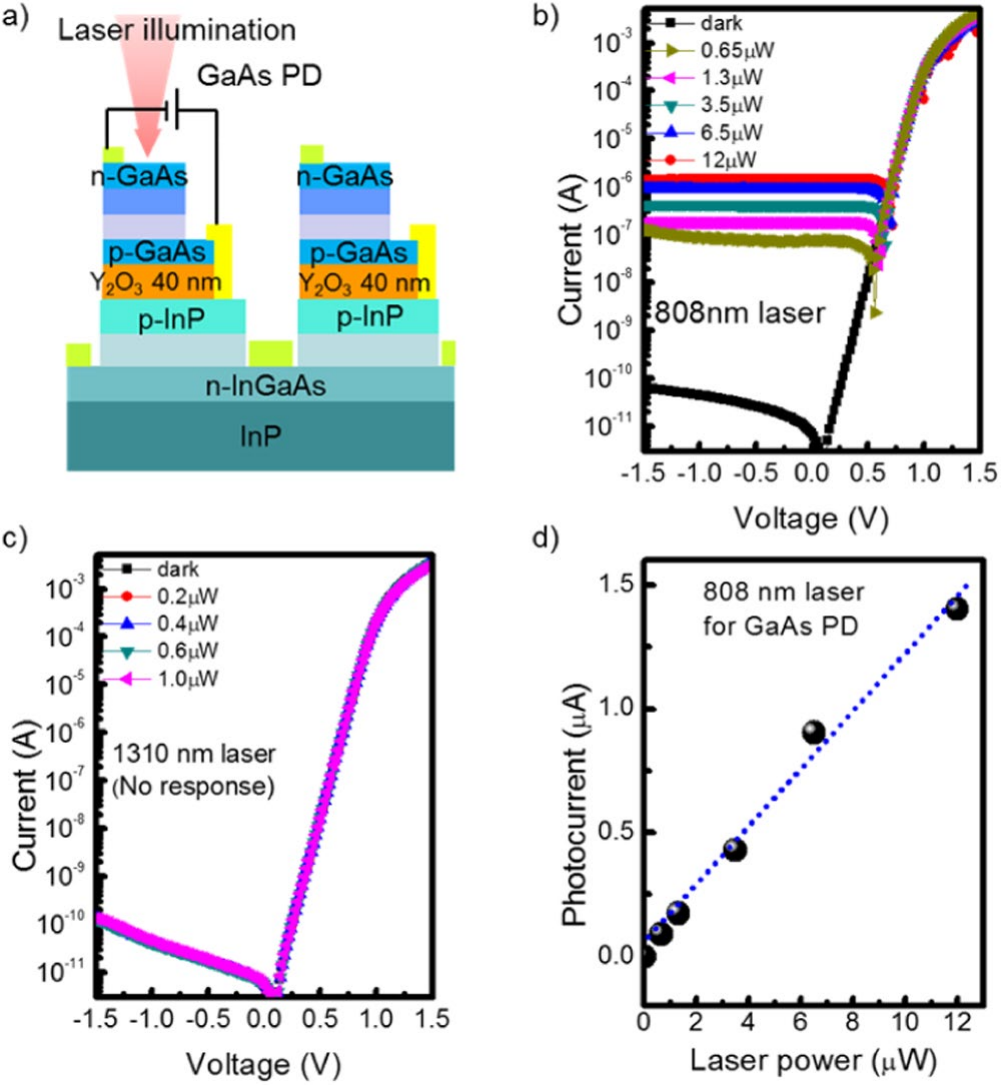
Investigation on the performances of GaAs top PD by using optical measurements. (**a**) Schematic of GaAs top PD measurement in electrical characterization, (**b**) photoresponses of GaAs top PD with 808 nm laser and (**c**) 1310 nm laser, (**d**) photocurrents as a function of incident laser powers with 808 nm laser.

We also investigated the optical properties of the fabricated multicolor PD by using various laser diodes, namely, 520 nm, 620 nm, 808 nm, and 1310 nm of a tunable laser with a light coupling through the lensed fiber in a Keithley 4200 probe station. To accurately characterize the device performance at each wavelength, all incident laser intensities were carefully calibrated. A Si photodiode (Thorlabs) and a Ge photodiode (818-IR/CN, Newport) were used for the calibration of the light intensity for the visible wavelength ranges including 520/620/808 nm and 1310 nm, respectively. The optical power meter had the resolution of a nanowatt unit. All lasers were vertically incident to the top surface of the multicolor PD. Figure 6(a) shows the schematic structures under measurement when the bias was applied to the GaAs top PD to individually evaluate the performance of each PD. Figure 6(b,c) present the optical characteristics of the GaAs top PD with 808 and 1310 nm wavelengths (520 nm and 620 nm responses not shown). While clear photoresponses were observed with incident laser powers of 0.65, 1.3, 3.5, 6.5, and 12 μW with the 808 nm laser, the 1310 nm laser could not generate any photo-carriers within the GaAs PD because the GaAs absorption band cannot trace such a long-wavelength due to its bandgap. Then, we calculated the optical responsivity (*R*_i_) at −1 V bias as

$${R}_{{\rm{i}}}={I}_{{\rm{ph}}}/{{\rm{P}}}_{{\rm{in}}}.$$

Regarding 520 nm and 620 nm wavelengths, the GaAs PD showed responsivity values of 2.2 mA/W and 40 mA/W, respectively. Also, the responsivity for the 808 nm wavelength was calculated to be 0.15 A/W. This GaAs top PD showed similar performance to that of the GaAs PD on Si with the same epitaxial structure reported in a previous paper^21^. The GaAs PD on Si showed responsivity of 0.06 A/W for the 635 nm wavelength. The origin oflow responsivity can be attributed to the free carrier absorption in the 600 nm-thick n^+^-GaAs layer and high reflectance at the top surface. Although the loss of incident light occurs, the performance of the top GaAs PD is comparable to that of the GaAs MSM PD in the multicolor structure in a previous study^12^. Finally, the power dependence of the photocurrents was measured for the 808 nm wavelength as shown in Fig. 6(d). The photocurrents linearly increased with increasing laser power, which indicates good linearity of the GaAs PD with incident radiation.

**Figure 7:**
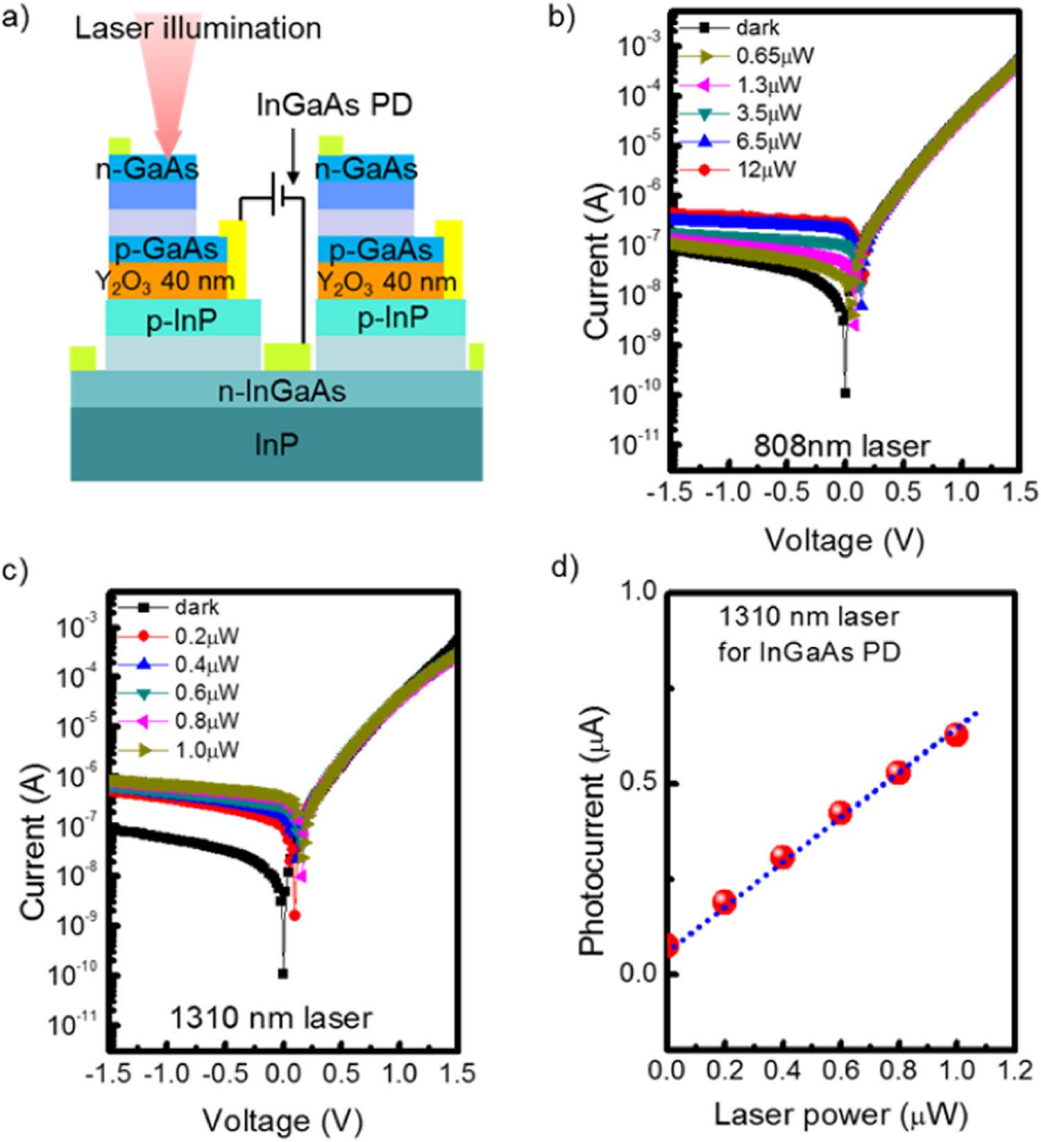
Investigation on the performances of InGaAs bottom PD by using optical measurements. (**a**) Schematic of InGaAs bottom PD measurement in electrical characterization, (**b**) photoresponses of InGaAs bottom PD with 808 nm laser and (**c**) 1310 nm laser, (**d**) photocurrents as a function of incident laser powers with 1310 nm laser.

Figure 7(a) shows the schematic structure of the InGaAs bottom PD in the optical measurement. The optical characteristics of the InGaAs bottom PD as a function of incident wavelengths of 808 nm and 1310 nm. When we illuminated the top GaAs PD surface with 808 nm lasers, weak photoresponse was observed with the same light powers in comparison with that of the GaAs PD as seen in Fig. 7(b). A responsivity of 30 mA/W was obtained under −1V bias, suggesting that the photoresponse could be attributed to the relatively small number of photons, which are unabsorbed photons in the GaAs PD. For 1310 nm laser illumination, the InGaAs PD showed a strong photoresponse despite relatively weak powers, such as 1 μW, compared with the 808 nm laser, as shown in Fig. 7(c). The strong photoresponse can be attributed to adjacent laser wavelengths of the InGaAs band edge. As a result, the responsivity at 1310 nm was found to be 0.47 A/W, which is 15 times higher than that at 808 nm. A 1310 nm wavelength can pass through the GaAs top PD region to the InGaAs bottom PD despite the GaAs top PD region with 1.85 μm. In addition, this responsivity value is similar to that of the reference InGaAs PD at 1310 nm wavelength, which was 0.53 A/W. This slight difference can be mainly attributed to the free carrier absorption in the doped layers and the internal reflection of layers with various refractive indexes other than phonon interaction with impurities, defects, and so forth in the GaAs top PD region^21–23^. As seen in Fig. 7(d), the InGaAs bottom PD showed good linearity for 1310 nm laser illumination, which also suggests good operability of InGaAs PD.

**Figure 8:**
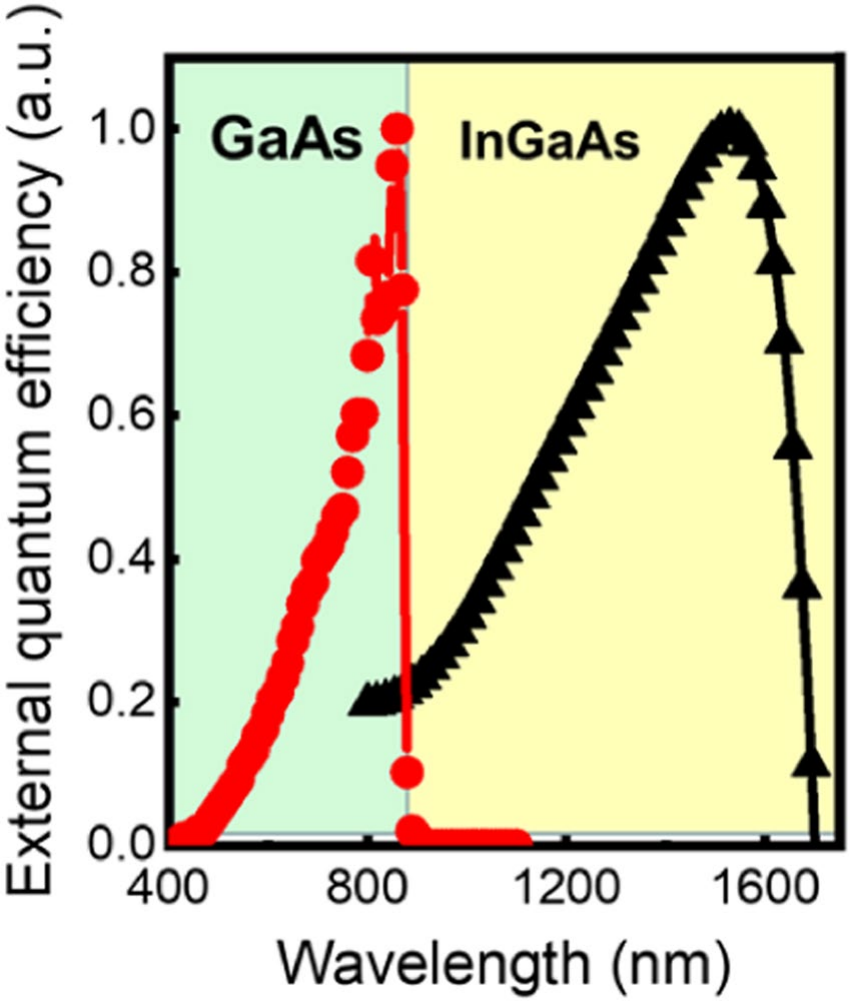
Measurement of spectral responses of formed multicolor PDs. The EQE spectrum of the fabricated GaAs PD and InGaAs PD in a multicolor structure as a function of the light wavelengths.

Figure 8 shows the external quantum efficiency (EQE) results of the GaAs and InGaAs p-i-n PD in multicolor PDs as a function of wavelength. The normalized EQE spectra were obtained using a K3100 Spectral IPCE Measurement System equipped with a 300 W Xe light source and monochromator (McScience, Korea). While the GaAs EQE was measured with the calibration source of a Si diode with a spectral range from 400 to 1100 nm, the InGaAs EQE was calibrated with an InGaAs diode with a spectral range from 800 to 1700 nm. Although the absolute EQE could not be extracted due to the limited capability of the measurement equipment, it was sufficient to show the wavelength coverage of each PD. The GaAs PD showed an EQE increase with increasing wavelength until the GaAs band edge. Thus, the maximum EQE value was observed at the GaAs band edge of 870 nm. The EQE of the InGaAs bottom PD also showed a similar trend to that of the GaAs PD. The shape of the EQE spectra can be attributed to the absence of anti-reflection coating (ARC) at the surface, resulting in the relatively low EQE at shorter wavelength regions in both PDs. The EQE values for shorter wavelength regions can be improved by the combination of proper ARC techniques. Nevertheless, these results clearly confirmed that the multicolor PD can cover the wavelength ranges from visible to near-IR in the material’s absorption regions, respectively. In addition, the calculated responsivity of the InGaAs bottom PD corresponds to approximately 45% quantum efficiency at 1310 nm. A quantum efficiency of 23% was obtained in the GaAs top PD at 808 nm from the responsivity results. These quantum efficiencies are significantly improved values in contrast to those of conventional multicolor PDs due to the high material quality and p-i-n structure^12^. Also, there is much room to further improve detector performance in terms of the design of layer thicknesses, ARC effect, and ohmic contact properties.

**Figure 9:**
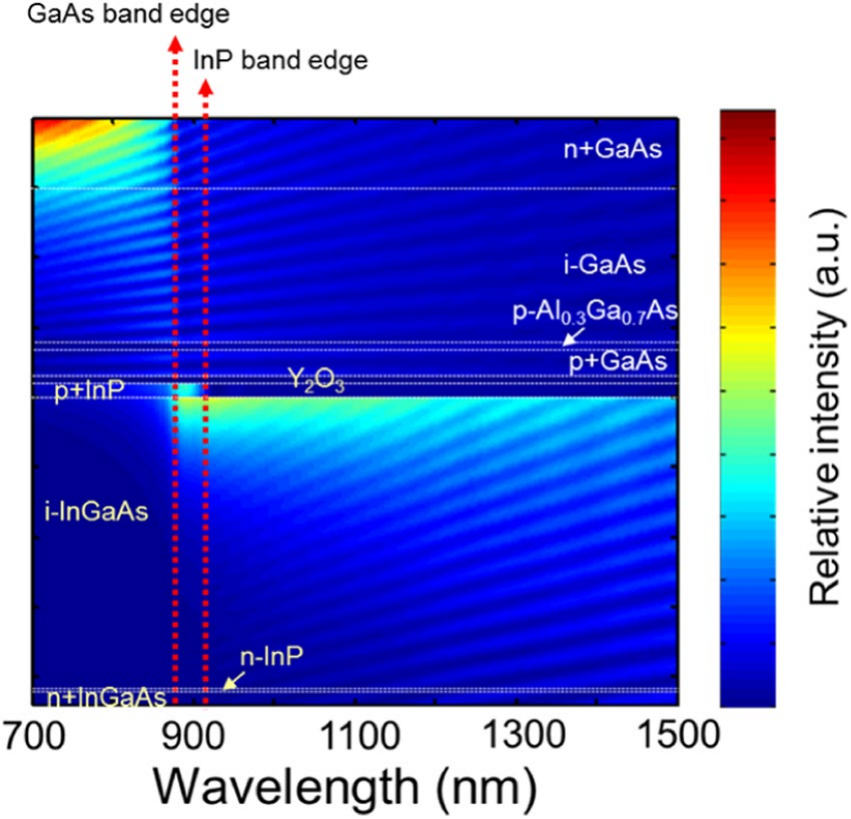
Simulation of optical intensity distribution for multicolor PD structure.

To investigate the absorption behavior of multicolor PDs, the optical intensity distribution in a multicolor PD structure was simulated as seen in Fig. 9. It shows the optical intensity distribution as a function of the wavelength of the incident light for the GaAs PD and the InGaAs PD with a Y_2_O_3_ bonding layer. Incident light below 870 nm of the GaAs absorption edge is considerably absorbed in the top PD region; a small amount of light could reach the intrinsic InGaAs region. Beyond the GaAs absorption edge, weak absorption was observed in the top p-InP region, which cannot contribute to the photocurrent. However, a long-wavelength region above 920 nm of the InP absorption edge is mainly absorbed in the intrinsic InGaAs region. Moreover, there is still a significant amount of passing light that reaches the n-InGaAs contact layer. It should be noted that there is a strong photoresponse at 1310 nm, which is definitely consistent with this intensity simulation. These results also suggest that enhancement of detector performance will be further optimized through layer thickness optimization and facilitation of ARC.

**Figure 10:**
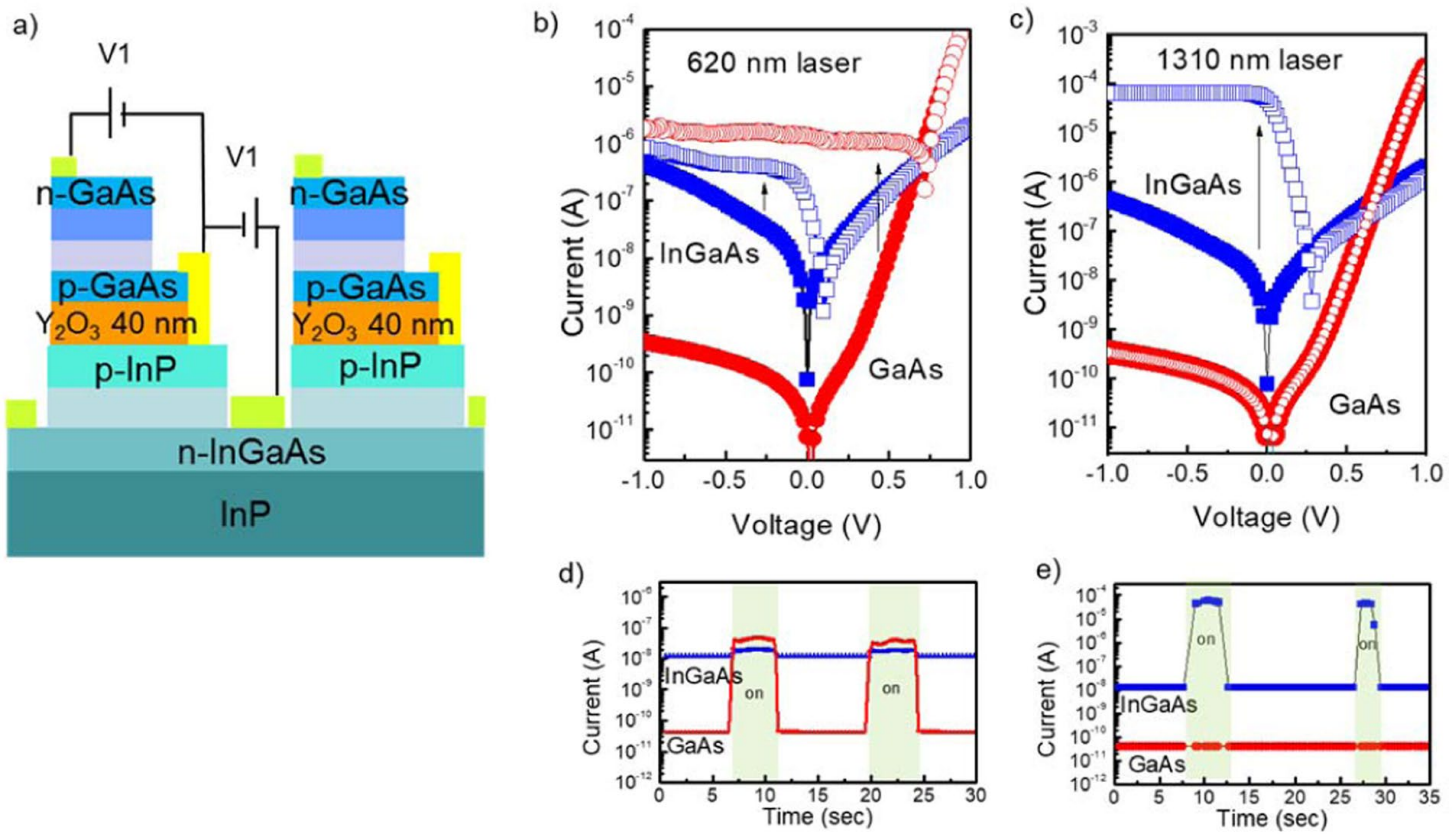
Investigation of simultaneous detection for GaAs top PD and InGaAs bottom PD with independently applying the bias (**a**) Schematic of GaAs top PD and InGaAs bottom PD measurement for simultaneous detection tests, (**b**) photoresponses of GaAs and InGaAs PD with 638 nm laser and (**c**) 1310 nm, (**d**) switching performances at −0.1 V with uncertain laser powers for 638 nm and (**e**) 1310 nm.

Finally, to verify the ability to detect the photons with different energies simultaneously, we carried out the measurement on two wavelengths sensing with sweeping the bias as shown in Fig. 10(a). Bias ranging from −1 V to 1 V was applied to the GaAs and InGaAs PDs at the same time with a p-doped layer as a common ground. Figure 10(b,c) show the photoresponses at 620 nm and 1310 nm, respectively. These measurements were obtained with unknown laser powers. While the solid and open symbol represent current under dark current (*I*_dark_) and the current under the illumination (*I*_illumination_). All top GaAs and bottom InGaAs PDs clearly generated photocurrents for 620 nm signal incident from a fiber. The *I*_illumination_/*I*_dark_ of GaAs PD showed 10^4^ orders of magnitude at −0.5 V, which can be clearly distinguished Compared with that of InGaAs PD at 620 nm wavelength. On the other hand, at 1310 nm, the GaAs PD could not detect photons, whereas the InGaAs PD showed a high *I*_illumination_/*I*_dark_ of 10^3^ orders of magnitude. Additionally, the switching characteristics of the multicolor PDs were demonstrated as shown in Fig. 10(d,e). When both PDs were measured at −0.1 V, the input signals were manipulated by on/off switching in terms of the incidence of different wavelengths. These results indicated that there is no mutual interference between the GaAs top PD and the InGaAs bottom PD in simultaneous detection because signals would be selectively processed in readout circuits (ROICs). Electrically independent access for two-color detection was successfully demonstrated by using monolithic integration.

Many researchers have reported various formation methods for implementing multicolor PDs, such as bulk epitaxy, adhesive bonding, transfer printing, and packaging, as shown in Table 1 ^8,12,13,24^. We evaluated five figures of merit of the PDs, including material quality, vertical alignment, pixel density, array formation, and freedom of wavelength selection. Even without the epitaxy method, previous bonding methods have disadvantages in terms of vertical alignment and array formation. Unlike these methods, superior characteristics for the increase of pixel density and array formation can be achieved in the proposed multicolor PDs. Additionally, an extra cost reduction could be obtained by using substrate recycling techniques^16^.

**Table 1:** Benchmark of multicolor PDs which ever reported.

	GaSb//InAsSb [8]	GaAs//InGaAs [12]	Si//InGaAs [13]	Si//InGaAs [24]	GaAs//InGaAs [This work]
Integration method	Bulk epitaxy	Adhesive bonding	Transfer printing	Packaging	High throughput ELO
Material quality	▲	●	●	●	●
Vertical alignment	●	▲	▲	●	●
Pixel density	●	●	▲	×	●
Array formation	●	▲	▲	×	●
Freedom of wavelength selection	▲	●	●	●	●

[Appropriate (●), weak (▲), inappropriate (×)].

## Conclusions

We have demonstrated the monolithic integration of visible GaAs p-i-n PDs with near-IR InGaAs p-i-n PDs by using a high-throughput ELO process. While the multicolor PDs showed vertically well-aligned of GaAs and InGaAs epitaxial layers confirmed by XRD measurement, a good bonding interface was also observed with no visible defects by TEM imaging. Excellent rectifying characteristics were shown with a 10^7^ and 10^4^ on/off ratio at ±1.5 V for the GaAs and InGaAs PDs, respectively. In optical characterization, photoresponse cannot be observed at 1310 nm, while the top GaAs photodetector showed responsivity of 0.15 A/W at 808 nm. The responsivities of 0.47 A/W at 1310 nm and 30 mA/W at 808 nm were obtained in the InGaAs bottom PDs. These photoresponses of multicolor PDs in the 700–1700 nm wavelength range were consistent with the simulation of the optical intensity distribution. These photoresponses suggest that integrated photodetectors are independently operated without interference between the top and bottom PDs. Based on these results, we believe that this method can be expanded from UV of GaN material to IR of InAsSb material to reduce the complexity of other approaches for multicolor PDs.
